# The complete mitochondrial and plastid genomes of *Rhododendron simsii*, an important parent of widely cultivated azaleas

**DOI:** 10.1080/23802359.2021.1903352

**Published:** 2021-03-24

**Authors:** Jie Xu, Hang Luo, Shuai Nie, Ren-Gang Zhang, Jian-Feng Mao

**Affiliations:** a Beijing Advanced Innovation Center for Tree Breeding by Molecular Design, National Engineering Laboratory for Tree Breeding, College of Biological Sciences and Technology, Beijing Forestry University, Beijing, China; b Beijing Ori-Gene Science and Technology Co. Ltd., Beijing, China

**Keywords:** *Rhododendron simsii*, azalea, mitochondrial genome, plastid genome

## Abstract

The genus *Rhododendron* of the heather family (Ericaceae) is well known and widely cultivated for their highly ornamental value. The most widely cultivated *Rhododendron* species is *Rhododendron simsii* (Indoor azalea). In this study, we assembled the complete linear mitochondrial genome (GenBank accession number MW030508) and quadripartite plastid genome (GenBank accession number MW030509). The mitochondrial genome is 802,707 bp in length with containing 53 unique genes (33 protein-coding, 17 tRNA, and 3 rRNA genes), while the 152,214 bp long plastid genome is smaller and containing 105 unique genes (4 rRNA, 26 tRNA, and 75 protein-coding genes). Phylogenetic analysis showed that the same species relationship with APG system as well as the low supports of branches which is the common characteristic of resolved Ericales phylogenetics.

The genus *Rhododendron* of the heather family (Ericaceae) is well known for the outstanding beauty and great diversity of corolla among its more than 1000 species and 30,000 cultivars (Stevenson 1930; Sleumer 1949; Galle 1985; Yan et al. 2015). *Rhododendron simsii* Planch. 1853. (Indoor azalea), is the most widely cultivated *Rhododendron* species (Ding 2009; Kobayashi et al. 2013; De Riek et al. 2018). As the primary ancestors of azalea cultivars, *R. simsii* was introduced into Europe in the 18th century from China and its breeding for ornamental use began in England and was well developed in Belgium (Galle 1985; De Keyser et al. 2010; De Riek et al. 2018). Then, *R. simsii* hybrids become one of the most important pot plants in Belgium, with an annual production of approximately 40 million pots (De Keyser et al. 2010). Now azalea cultivars have become one of the most popular pot plants and landscape shrubs in Europe, North American, and Asia (Galle 1985; De Riek et al. 2018). Here, we determine mitochondrial and plastid content and structure of *R. simsii* through whole-genome sequencing, as well as detected its identity and phylogenetic relationship among Ericales. This study could bring more desirable information for evolutionary and functional studies in the future.

Leaves were obtained from a 20-year-old shrub from Jingshan, Hubei Province, China. Total DNA was isolated and extracted from the leaves using the DNeasy Plant Mini Kit (QIAGEN, Inc.) and then purified using the Mobio PowerClean Pro DNA Clean-Up Kit (MO BIO Laboratories, Inc.). A specimen was deposited at the Herbarium of Beijing Forestry University (http://www.bjfu.edu.cn/, Jian-Feng Mao jianfeng.mao@bjfu.edu.cn), under the voucher number mao_20190311.

For PacBio SMRT (single-molecule real-time) sequencing, libraries with 20-kb DNA inserts were prepared and sequenced on a PacBio RSII platform using P6-C4 chemistry (6 SMRT cells). A total of 6.5 million PacBio long reads were generated, yielding 51.15 Gb (roughly 100× coverage) with an average read length of 7705 bp. For Illumina sequencing, 150-bp paired-end (PE) libraries were constructed for sequencing on an Illumina HiSeq X Ten platform. Finally, ∼91.49 Gb (roughly 170× coverage) of raw sequencing data were obtained.

Preceding the filtered and corrected PacBio reads, genomic reads were mapped on both organelle genomes of closely related species by minimap2 v2.11-r797 (Li 2018); *Vaccinium macrocarpon* (NC_023338.1), *Rhazya stricta* (NC_024293.1), *Hesperelaea palmeri* (NC_031323.1)*, Corchorus capsularis* (NC_031359.1), and *Vitis vinifera* (NC_012119.1) for mitochondrial assembly; and *Cymbidium ensifolium* (NC_028525.1), *Diospyros kaki* (NC_030789.1), *Pouteria campechiana* (NC_033501.1), *Diospyros blancoi* (NC_033502.1), and *Vaccinium macrocarpon* (NC_019616.1) for plastid assembly. All mapped reads were extracted for the following assemblies. Firstly, we used Canu v1.7 (Koren et al. 2017) and SMARTdenovo v1.0.0 (https://github.com/ruanjue/smartdenovo) (Liu et al. 2020) to generate two primary assemblies. For the plastid genome, the assembly from SMARTdenovo was selected for high quality by checking the continuity of the assembly. Similarly, contigs from Canu were used to assemble the mitochondrial genome using SeqMan v11 (Swindell and Plasterer 1997). Finally, we annotated and illustrated the two organelle genomes using the OGAP pipeline (https://github.com/zhangrengang/OGAP). Within the pipelines, Exonerate v2.2.0 (Slater and Birney 2005) and AUGUSTUS v3.3.1 (Stanke et al. 2006) were employed for identity protein-coding genes, tRNAscan-SE v2.0.5 (Lowe and Eddy 1997) and BALT v36 (Kent 2002) were used to confirm tRNA and rRNA respectively.

Finally, the mitochondrial genome gave a linear scaffold of 802,707 bp, with 45.87% GC content. A total of 53 unique genes, consisting of 33 protein-coding, 17 tRNA, and 3 rRNA genes. Among these genes, eight protein-coding genes (*atp*4, *ccmB*, *ccmC*, *mttB*, *nad*4*L*, *nad*9, *rpl*10, *rps1*), one tRNA genes *(trnW-CCA-cp*) and one rRNAgene (*rrn*26) have a duplicated copy, *trnM-CAT-cp* gene has three copies and *trnM-CAT* gene has four copies. In addition, *nad1* and *nad2* gene was trans-spliced. Comparatively, the 152,214 bp long plastid genome is much smaller, with a much lower GC content of 35.74%. Moreover, this plastid genome has the quadripartite structure found in most land plant plastid genomes, containing 105 unique genes (including 4 rRNA, 26 tRNA and 75 protein-coding genes). All the protein-coding genes and rRNA genes are single copy, with the exception of *trnI-CAU*, *trnM-CAU* and *trnV-UAC* gene which occur in quadruplicated, triplicated and duplicated copy, respectively. Besides, *rps12* gene was trans-spliced.

Phylogenetic analysis was performed with the single-copy genes of plastid genomes from *R. simsii* and other 28 plant species in the Ericales order. MAFFT v7.471(Katoh et al. 2002) was used to prepare sequences alignment which then was trimmed with trimAl v1.4.rev15 (Capella-Gutiérrez et al. 2009). Finally, the phylogenetic analysis was executed by IQ-TREE v2.0.3 (Nguyen et al. 2015) with the model of GTR + F+R2 and 1000 bootstrap replicates. As expected, the resolved topology was consistent with that of the APG (Angiosperm Phylogeny Group, http://www.mobot.org/MOBOT/research/APweb/) (Chase et al. 2016), and the low supports were observed for some branches, in consistent with the previously reported common characteristic of the Ericales (Rose et al. 2018; Larson et al. 2019) (Figure 1).

**Figure 1.**
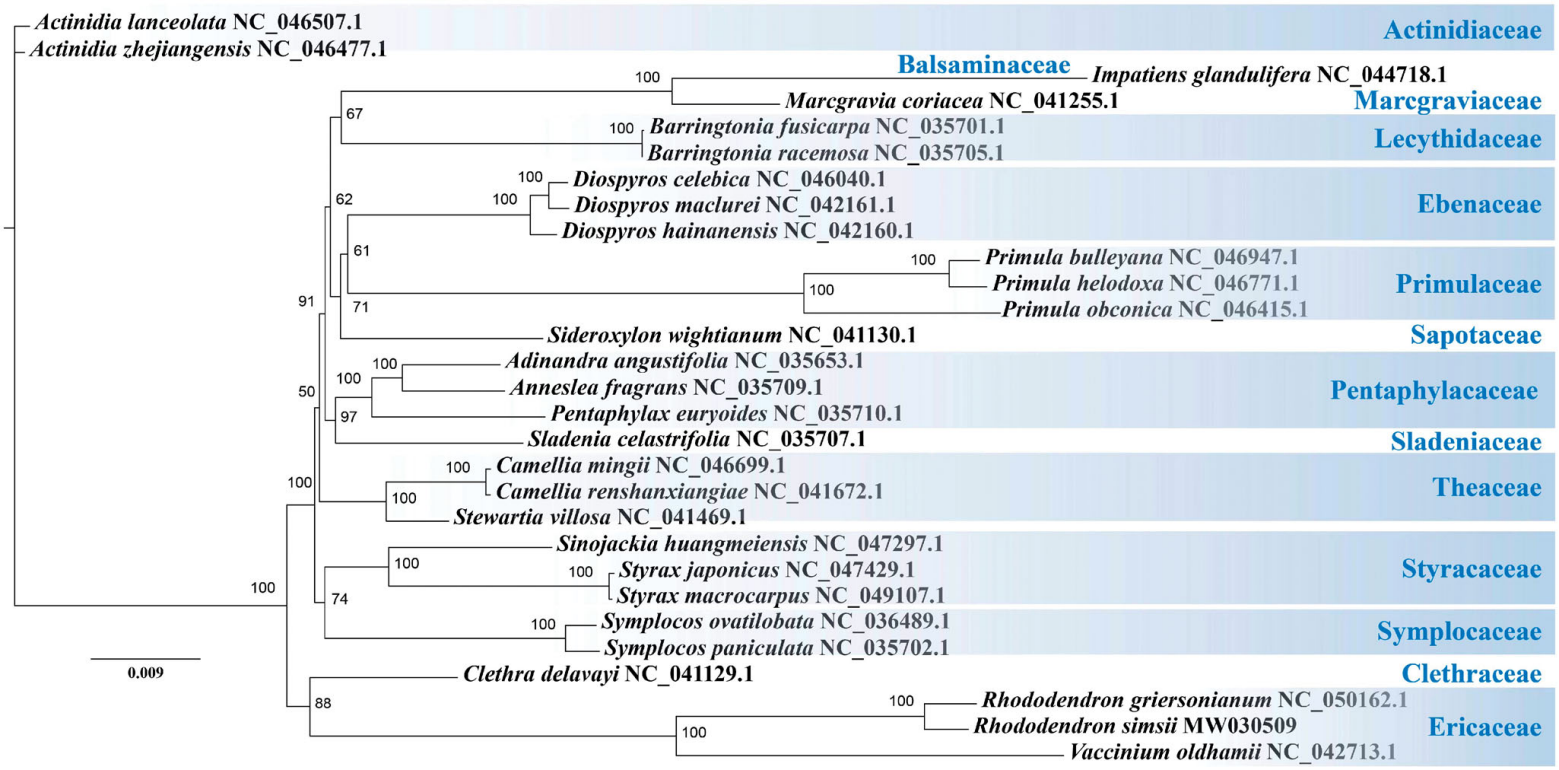
The maximum-likelihood tree based on single-copy genes from plastid genome of *R. simsii* and other 28 Ericales species. The numbers on the nodes indicate bootstrap values from 1000 replicates.

## Data Availability

The genome sequence data that support the findings of this study are openly available in GenBank of NCBI at (https://www.ncbi.nlm.nih.gov/) under the accession no. MW030508 (mitochondrial genome) and MW030509 (plastid genome). The associated BioProject, SRA, and Bio-Sample numbers are PRJNA588298, SRS5624703, and SAMN13241185, respectively.
